# Vaccine Beliefs Among Uninsured People Receiving Care at Free Clinics

**DOI:** 10.1007/s10900-024-01416-8

**Published:** 2024-10-22

**Authors:** Caroline C. Liu, Julio A. Siliezar, Omar Alzayat, Carly A. Robinson, Timothy Do, Adrianna I.J. Carter, Christine N. Pons, Om Patel, Michael S. Wilkes

**Affiliations:** 1 School of Medicine, University of California Davis, Sacramento, CA USA; 2 Department of Internal Medicine, University of California Davis, Sacramento, CA USA

**Keywords:** Vaccination, Free Clinic, COVID-19, Influenza, Perceptions of Vaccines

## Abstract

**Background:**

Misinformation and vaccination hesitancy contribute to disparities in vaccination rates, particularly in under-resourced communities. This study aims to investigate perceptions and factors influencing vaccination decisions at free clinics serving diverse, under-resourced communities.

**Methods:**

Surveys were conducted across eight free clinics in the Greater Sacramento area, targeting uninsured or underinsured individuals. Information on demographics, sources of vaccine information, access to vaccines, vaccine perceptions, and vaccination decisions as pertaining to influenza and COVID-19 were collected on Qualtrics software. Chi-square and t-tests were used to analyze associations between demographics and vaccination rates.

**Results:**

Among 109 participants (24–82 years old), vaccination rates were found to be higher than the county average, with notable demographic variations. Contrary to initial hypotheses, men had higher vaccination rates than women, and recent immigrants exhibited higher vaccination rates than more long-term U.S. residents. A higher number of participants regarded the COVID-19 vaccine as effective than as safe, while the reverse was true for the influenza vaccine. Healthcare providers were the most trusted and influential sources for vaccine information, followed by government agencies, and then family and friends. Answers to hypothetical vaccine scenarios elicited assessments on risks and benefits.

**Conclusion:**

The study provides insight into the dynamics of vaccine hesitancy and factors that play into the decision-making process in under-resourced communities, underscoring the role of trust in healthcare providers. These findings are vital for tailoring community outreach strategies to create trust, address barriers, and enhance vaccine uptake within free community clinics.

## Introduction

The toll of COVID-19 in the U.S. has been severe, with 1.19 million deaths as of June 22, 2024 [1]. Not only did the pandemic affect the physical health of those who contracted the virus, but it also detrimentally impacted the mental health and well-being of the public, shaking their trust in the public health system [2]. By December of 2020, two American vaccines were developed and approved by the Food and Drug Administration (FDA) for emergency use; however, logistical challenges, like deploying the vaccine to the public and widespread vaccine misinformation, remained as barriers towards vaccination [3].

The World Health Organization (WHO) identifies vaccine hesitancy as one of the top 10 threats to global health [4]. Despite strong evidence of vaccine safety and efficacy [5–8], levels of vaccine hesitancy continue to increase [9]. The WHO EURO Vaccine Communications Working Group proposed a model with three determinants contributing to vaccine hesitancy: convenience, confidence, and complacency [10]. Convenience includes factors such as availability, affordability, accessibility, literacy, and quality of service. Confidence relates to trust in vaccine effectiveness or safety, healthcare system reliability, and government motives. Complacency emerges when disease risk is perceived as low compared to vaccination risk. Misconceptions of a low risk-to-benefit ratio have been attributed as a primary factor in low vaccination rates [11, 12]. Additional factors that precipitate vaccine hesitancy include safety concerns and skepticism towards vaccine effectiveness [13, 14]. For example, media-driven escalation of conspiracy theories, misinformation, and misperceptions regarding vaccination side-effects fuel skepticism and contribute to negative public perception [15–18].

Prior studies indicate that social determinants influence vaccination status, including education, financial resources, healthcare access, cultural upbringing, and community relationships [19]. These factors impact people’s trust and feelings towards vaccines. Economic instability and lower educational attainment significantly correlate with vaccine hesitation and refusal [20, 21]. In urban settings, many neighborhoods are severely under-resourced from a health perspective. Population-level immunity (herd-immunity) necessitates vaccinating 70–85% of the population; therefore, low vaccination rates can impede herd immunity and place communities at risk for infectious diseases [22].

In Sacramento County, the COVID-19 vaccination rate is 77% (based on most recent available data from December 2022), which is lower than the California average of 85% [23]. In the city of Sacramento, 11 student-run clinics provide free health care services to those in need. These 11 university-affiliated free clinics work together to provide culturally responsive care to uninsured or underinsured individuals, many of whom are immigrants. The majority of the patients are from under-resourced communities and lack the financial means and/or health literacy to access affordable healthcare.

This study explores the gaps in vaccination among diverse populations attending these clinics in effort to address any barriers in vaccine access. The primary objective is to investigate sources of vaccine information, trust, perceptions of effectiveness, access to, and factors influencing vaccine decisions within a diverse community of socioeconomically disadvantaged people attending free clinics. Specifically, we focus on perceptions of COVID-19 and influenza while utilizing hypothetical vaccine scenarios to elicit risk-benefit decisions. Our secondary aim is to draw correlations between vaccination rates and demographic factors in our patient population. Our hypothesis in relation to demographic factors include the following: women will have a higher vaccination rate than men, Asian participants will have the highest rates of vaccination, more recent immigrants to the US will have a higher vaccination rate, married participants will be more likely to be vaccinated than unmarried participants, and those with children will be more likely to be vaccinated.

## Methods

All 11 clinics were invited to participate in this study, with eight having adequate capacity to participate. Each clinic had a medical student lead and undergraduate volunteers for study-related tasks. Enumerators, trained in obtaining informed consent and survey administration, enrolled participants during clinic hours of operation between September 24, 2022 and February 12, 2023. The enumerators collected survey data with Qualtrics software using laptop computers or tablets.

All efforts were made to ensure the privacy and confidentiality of study participants, who provided verbal consent to participate in the study. Demographic data such as age, nationality, and duration of time lived in the U.S. were collected without names, birthdates, or other unique identifiers. Consent documents and survey materials were made available in English, Spanish, Vietnamese, and Cantonese. Participants were informed of confidentiality measures and the right to withdraw participation and received printed consent forms in their preferred language. Surveys were verbally interpreted, with questions and responses exchanged in English to the participant’s preferred language. Exclusion criteria included prior survey completion, inability or unwillingness to consent, individuals under 18 years of age, and participants who were not able to access the survey in any of the languages offered. All enrollment documents were encrypted and accessible only by study personnel.

If participants were unable to complete a written survey, enumerators offered to administer it orally and marked participant responses on their behalf. Statistical Analysis Software was used for analysis, collectively treating all data from participating clinics due to the small sample size. Demographic results were aggregated based on whether participants had received at least one dose of the COVID-19 vaccine. Unequal variance two sample t-tests and chi-square *p*-values were computed for associations.

## Results

Consent was provided by 130 participants across eight of the 11 free clinics. The survey was completed by 109 people whose answers are included in this analysis. The remaining 21 surveys were excluded due to incompleteness. Vaccinated patients refer to those who have received at least one dose of any COVID-19 vaccine and unvaccinated patients refer to those who have not received any doses. All participants considered one of the clinics as the site where they opted to receive their primary care.

Demographic data is summarized based on whether participants received at least one dose of a COVID-19 vaccine (Table 1). The average age of participants was 53 years. Vaccination rates were higher in males than females (93% vs. 86%). Overall vaccination rates were high, with 86% of Latino, 85% of Black/African American, and 100% of Asian participants having received at least one COVID-19 vaccine dose. Among those born in or residing in the U.S. for over a decade (73% of participants), the vaccination rate was 87%. In contrast, individuals residing in the U.S. for less than 10 years had a higher vaccination rate at 93%. Vaccination rates were higher among married individuals (91%) compared to unmarried (85%). Additionally, 84% of patients with children and 93% without children reported being vaccinated.

**Table 1:** Demographic data of participants by COVID-19 vaccination status

	Total	Not vaccinated	Vaccinated	*P*-value
	*N* = 109	*N* = 12	*N* = 97	
Age, *n* (%)				0.05951
Mean (SD)	52.9 (12.58)	46.5 (10.58)	53.6 (12.63)	
Median (IQR)	53.5 (46.5, 61.0)	49.0 (43.0, 56.0)	55.0 (47.0, 63.0)	
Range	24.0, 82.0	27.0, 58.0	24.0, 82.0	
Gender, *n* (%)				0.50372
Male	43 (39%)	3 (25%)	40 (41%)	
Female	65 (60%)	9 (75%)	56 (58%)	
Non-binary	1 (1%)	0 (0%)	1 (1%)	
Race, *n* (%)				0.29232
Latino	64 (59%)	9 (82%)	55 (57%)	
Black	13 (12%)	2 (18%)	11 (11%)	
Asian	21 (19%)	0 (0%)	21 (22%)	
White	6 (6%)	0 (0%)	6 (6%)	
Other	4 (4%)	0 (0%)	4 (4%)	
Born in US, *n* (%)				0.61052
No	75 (73%)	8 (67%)	67 (74%)	
Yes	28 (27%)	4 (33%)	24 (26%)	
Education level, *n* (%)				0.49362
None	9 (8%)	0 (0%)	9 (9%)	
Some	61 (56%)	8 (67%)	53 (55%)	
College graduate	39 (36%)	4 (33%)	35 (36%)	
Housing, *n* (%)				0.50262
No	16 (15%)	1 (8%)	15 (16%)	
Yes	92 (85%)	11 (92%)	81 (84%)	
Marriage, *n* (%)				0.31512
No	47 (45%)	7 (58%)	40 (43%)	
Yes	58 (55%)	5 (42%)	53 (57%)	
Children, *n* (%)				0.14002
No	56 (53%)	4 (33%)	52 (56%)	
Yes	49 (47%)	8 (67%)	41 (44%)	
Income, *n* (%)				0.27462
< 50 K	70 (76%)	9 (90%)	61 (74%)	
>=50 K	22 (24%)	1 (10%)	21 (26%)	

**Fig. 1:**
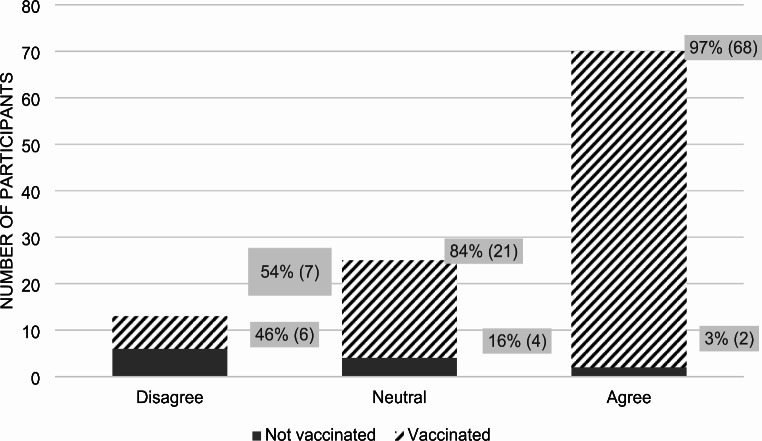
Participant answers to the statement: “I think the COVID-19 vaccines are safe.”

Of the 109 patients, 89% received at least one COVID-19 vaccine dose, with 64% having received three or more doses including the initial booster. Overall, 65% considered the vaccine safe, while 12% deemed it unsafe (*p*-value < 0.0001). Among those who agreed with the statement, “I think the COVID vaccines are safe,” 97% were vaccinated against COVID-19, and 3% were not. Among those who disagreed with the statement, 54% were vaccinated and 46% were not (Fig. 1).

**Fig. 2:**
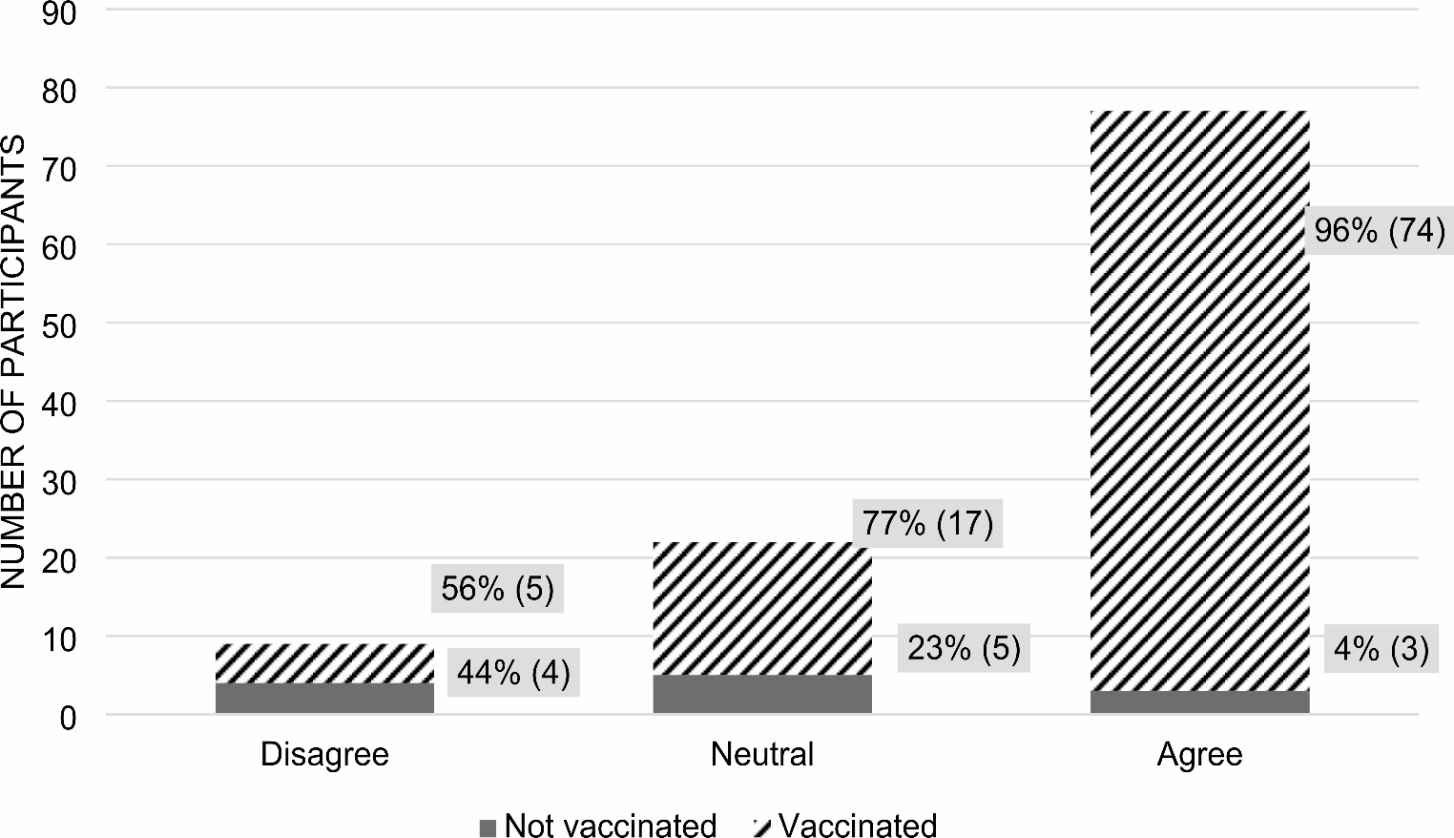
Participant answers to the statement: “I think the COVID-19 vaccines are effective.”

**Fig. 3:**
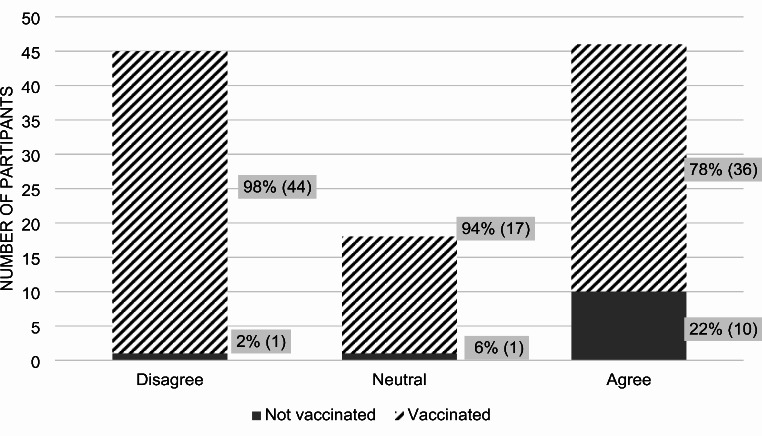
Participant answers to the statement: “I am/was hesitant to receive the COVID-19 vaccine.”

71% of participants believed in the COVID-19 vaccine’s effectiveness (*p*-value 0.0002). Among those who agreed, 96% were vaccinated against COVID-19 and 4% were not (Fig. 2). In terms of vaccine hesitancy, defined by uncertainty in their choice to receive the vaccine, 42% of all participants admitted to being hesitant at any point, while 41% did not (*p*-value 0.0087) (Fig. 3). Out of those who expressed hesitancy, 78% received the vaccine and 22% did not.

**Fig. 4:**
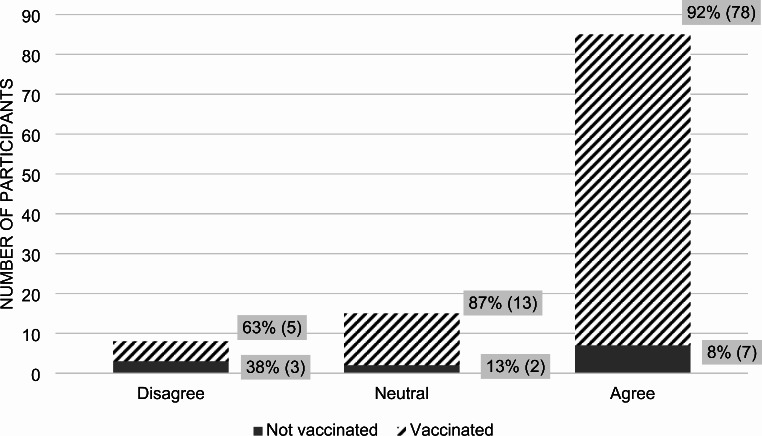
Participant answers to the statement: “I think the influenza vaccine is safe.”

We compared attitudes towards the COVID-19 vaccine with that of the influenza vaccine. In total, 79% of all participants agreed that the annual influenza vaccine is safe, while 7% disagreed (*p*-value 0.0402) (Fig. 4). Out of those who regarded the influenza vaccine as safe, 92% were vaccinated against COVID-19 while 8% were not. Out of those who regarded the influenza vaccine as unsafe, 63% were vaccinated against COVID-19 while 38% were not.

**Fig. 5:**
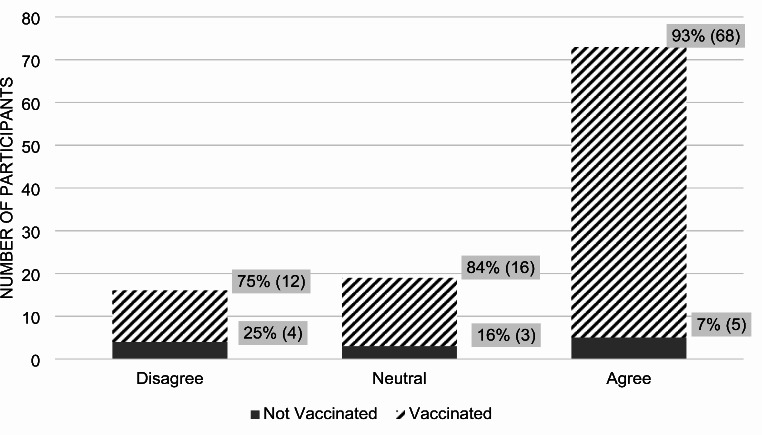
Participant answers to the statement: “I think the influenza flu vaccine is effective.”

Regarding effectiveness, 68% believed the influenza vaccine is effective, while 15% deemed it ineffective (*p*-value 0.0868) (Fig. 5). Out of the 68% who thought it was effective, 93% received the COVID-19 vaccine and 7% did not. Out of the 15% who disagreed and thought it was ineffective, 75% received the COVID 19 vaccine and 25% did not.

**Fig. 6:**
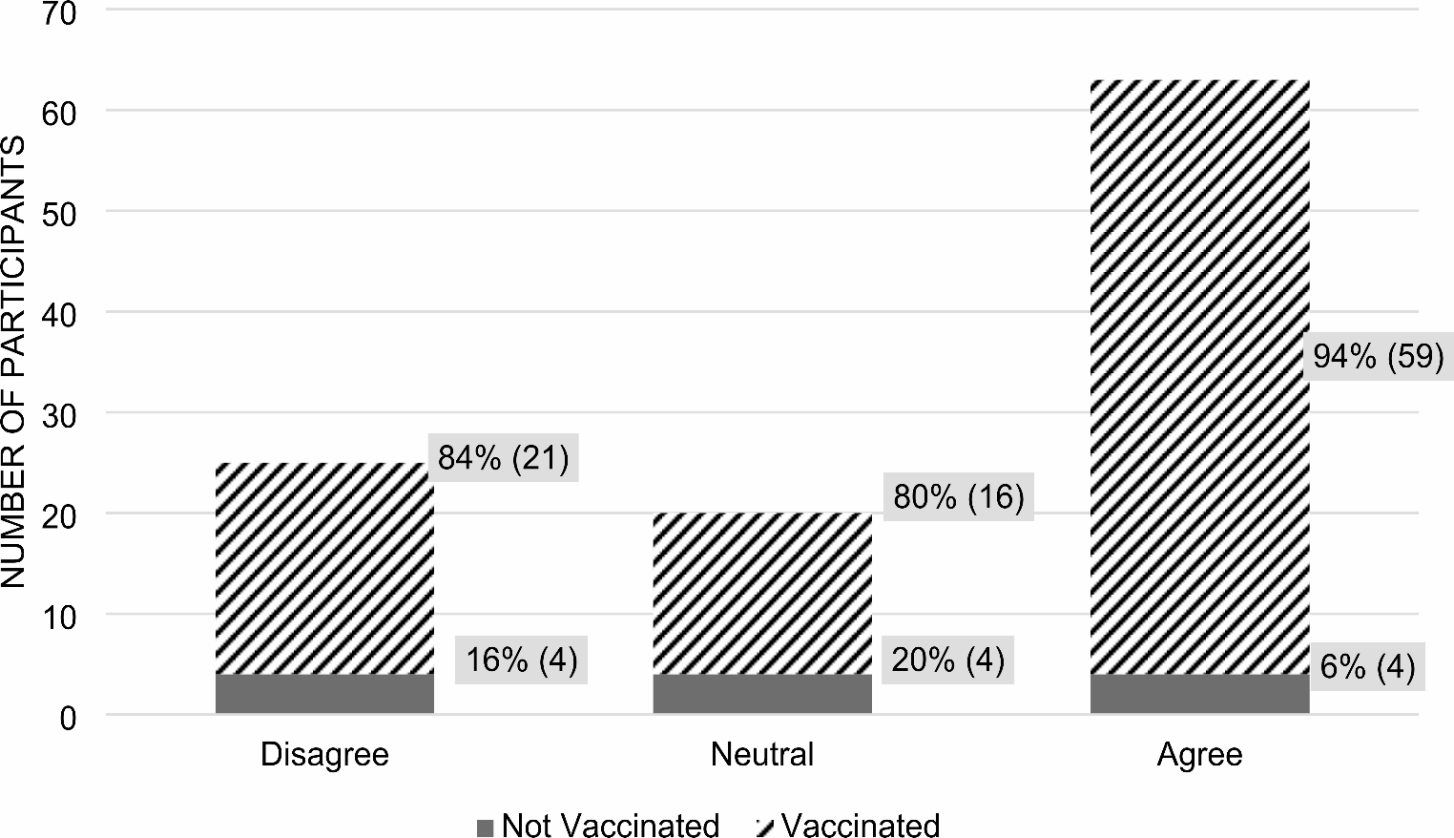
Participant answers to the statement: “Getting the influenza vaccine is important to me.”

Overall, 58% of total participants deemed it important to obtain the annual influenza vaccination, while 23% considered it not important (*p*-value 0.1611) (Fig. 6).

**Fig. 7:**
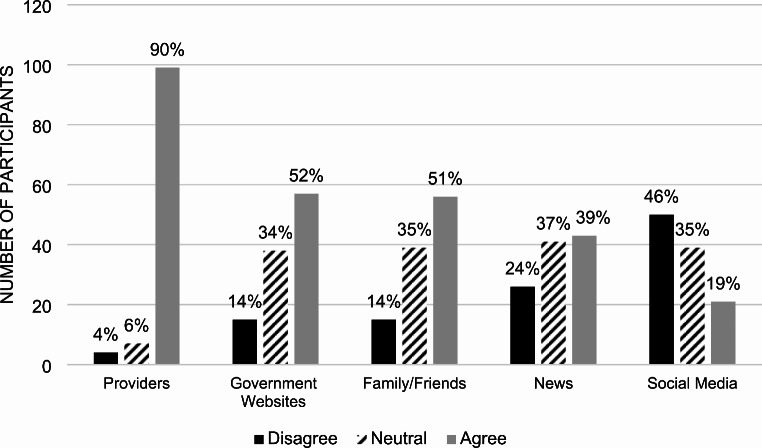
Participant answers to the statement: “I trust the following sources of information about vaccines.”

Participants ranked healthcare providers as the most trusted source of information when making vaccine decisions, with 90% of participants considering them trustworthy (*p*-value 0.0099) (Fig. 7). Government-sponsored sources (e.g., Centers for Disease Control and Prevention [1]) and family and close friends came second and third, with 51% and 52% of participants trusting them, respectively. Social media outlets were the least trusted, with only 19% of participants relying on them for vaccine information.

Patients’ primary reasons for COVID-19 vaccination were to protect themselves, their families, and their friends, while concerns about vaccine safety deterred some. For the influenza vaccine, perceived ineffectiveness was the main reason participants chose not to receive the vaccine. Overall, patients balanced protection, safety, and efficacy when making vaccination decisions.

**Fig. 8:**
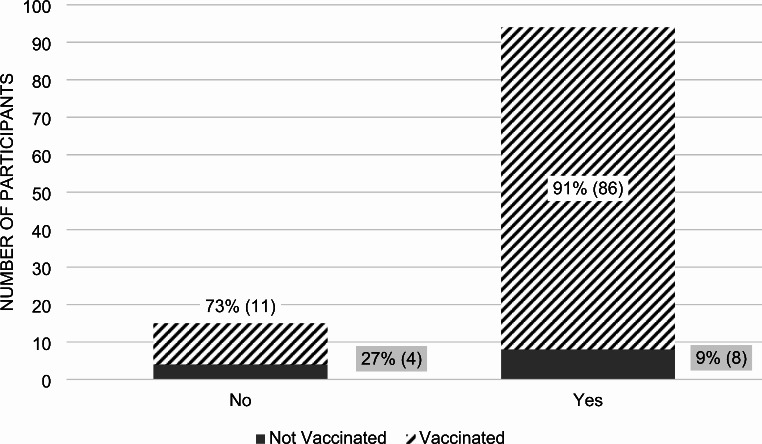
Vaccination Decisions for Hypothetical 1: A rare, but deadly illness with 50% mortality “Yes” includes 86% of all participants who took the survey. “No” includes the remaining 14% of all participants

**Fig. 9:**
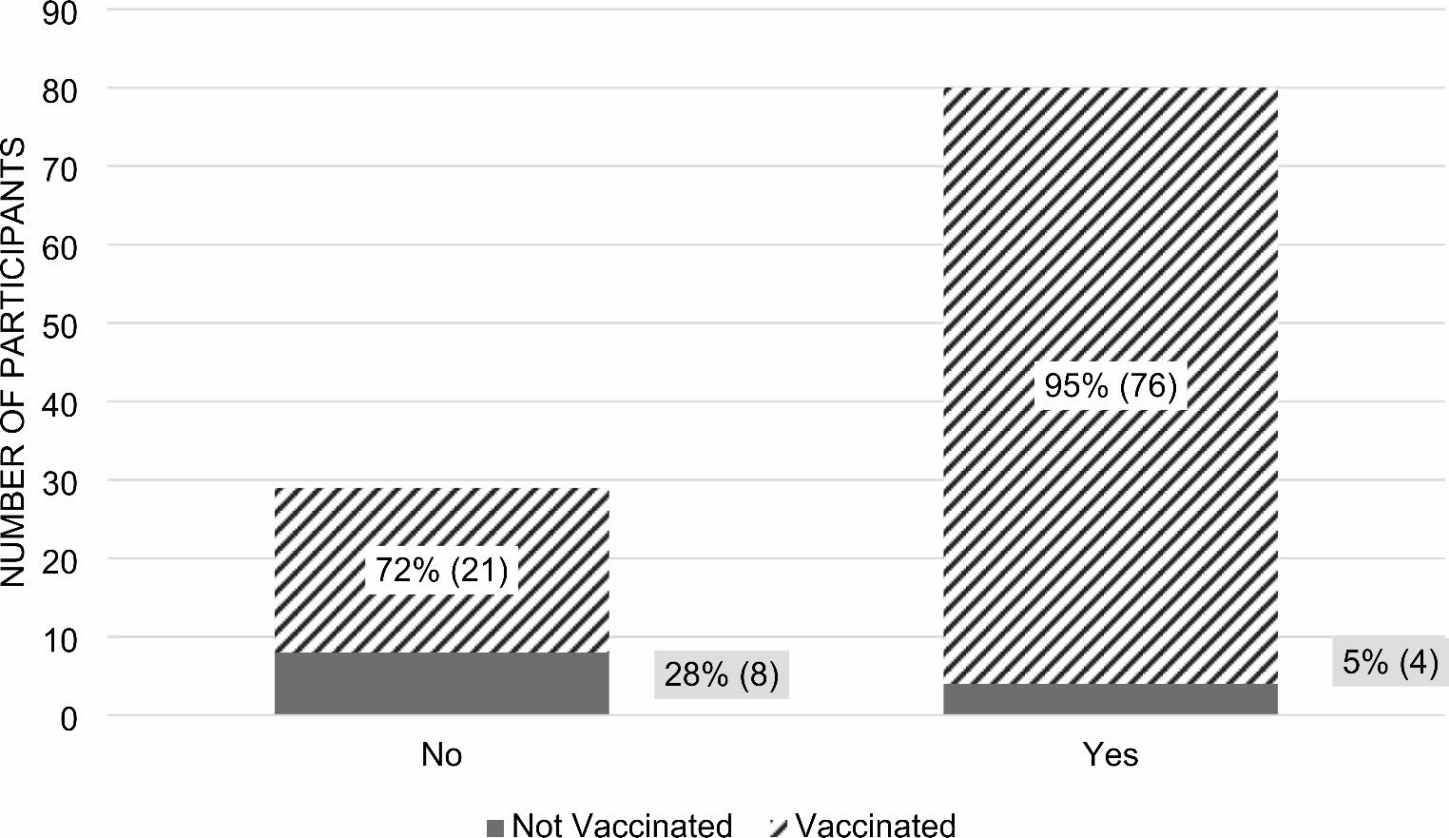
Vaccination Decisions Hypothetical 2: A more common, and less deadly illness with 1% mortality “Yes” includes 73% of all participants who took the survey. “No” includes the remaining 27% of all participants

We asked two hypothetical vaccine scenarios to help determine how participants weighed the risk of infection against the benefits of vaccination. In the first scenario, involving a “rare” disease with a 50% mortality rate, 86% of all participants expressed willingness to obtain the hypothetical vaccine (*p*-value 0.0370) (Fig. 8). Among those that said yes, 91% were vaccinated against COVID-19, and among those that said no, 73% were vaccinated. In the second scenario, featuring a “common” disease with a 1% mortality rate, 73% of participants stated they would obtain the hypothetical vaccine (Fig. 9).

## Discussion

The findings of this study provide important insights into the factors influencing COVID-19 vaccination and general attitudes towards vaccines among a socioeconomically disadvantaged and culturally diverse population attending free clinics.

The overall COVID-19 vaccination rate for the clinics in this study is 89%, higher than Sacramento County’s vaccination rate of 77%. This reflects robust and effective public health efforts and vaccine accessibility within the populations served at free clinics. However, several barriers and determinants were identified that contribute to vaccine hesitancy or acceptance.

### High vaccination rates within specific demographic groups

Contrary to our hypothesis, men had a higher vaccination rate (93%) than women (86%). These results were echoed in a systematic review and meta-analysis, with most studies reporting men having higher rates of vaccination and higher intentions of getting vaccinated against COVID-19 [24]. Another study suggested that more women than men may perceive the COVID-19 vaccines as unsafe, ineffective, and having more risks than benefits, which could help explain the disparity seen in our study [25].

In accordance with our hypothesis, vaccination rates were notably high among Asian participants (100%), and slightly lower among Latino (86%) and Black/African American (85%) participants. These differences may be influenced by cultural factors, targeted vaccination campaigns and prior positive or negative experiences with the healthcare system [26, 27]. Interestingly, individuals residing in the U.S. for less than 10 years had a higher vaccination rate (93%) compared to those who have been in the U.S. longer than ten years (87%), suggesting that recent immigrants might have higher trust in the healthcare system and vaccination campaigns in the U.S., or they might have faced more severe health challenges in their countries of origin, making them more receptive to vaccination through targeted initiatives [28].

As hypothesized, our data suggests that marriage status impacts COVID-19 vaccination status, with married participants being slightly more likely (91%) to be vaccinated than unmarried participants (85%). It is well known that spouses influence their partner’s healthcare behaviors [29], noted in a survey of 1,305 people living with their partners which found that couples’ COVID-19 vaccination statuses were 84.37% concordant [30]. In our study, having children seemed to be negatively correlated with participant vaccination rates, contrary to our hypothesis. Many nuances play into a parent’s decision to vaccinate their children versus themselves [24].

These differences noted within our patient population, including differences in vaccination rates between self-reported genders, racial backgrounds, length of time residing in the US, marriage status, and having children can be further supported through additional investigation. Understanding demographic disparities will help each clinic target care.

### Perceptions of COVID-19 vaccine safety, effectiveness, and hesitancy

Trust in the healthcare system and perception of vaccine safety and effectiveness were pivotal in vaccination decisions. Those who perceived the COVID-19 vaccine as safe (65%) and effective (71%) were significantly more likely to be vaccinated. More individuals believe the COVID-19 vaccine is “effective” than “safe” within both the vaccinated and unvaccinated groups. In addition, vaccine hesitancy remains a significant challenge, with 42% of participants admitting to being hesitant at some point likely due to concerns about vaccine safety, skepticism fueled by misinformation, and the perceived ineffectiveness of vaccines. In one study looking at reasons for COVID-19 vaccine hesitancy among Spanish-speakers in a free clinic in Ohio, the primary reasons for not vaccinating were fears of side effects or becoming sick from the vaccine [31]. This underscores the critical role of trust and accurate information in promoting vaccination.

### Comparison with influenza vaccination

In contrast, more individuals deem the influenza vaccine “safe” than “effective,” perhaps because the influenza vaccine has been available longer than the COVID-19 vaccine and patients are more familiar with it, giving it a perception safety. However, since the public is largely aware that one can still contract influenza after being vaccinated, it may lack the perception of effectiveness. These results likely contribute to patients being less hesitant, but still not choosing to obtain the vaccine due to perceived ineffectiveness. These results are consistent with a previous survey of influenza vaccine perceptions, where most adults reported the vaccine to be safe (86.3%) and a majority, but fewer considered it effective (73%) [32]. Regardless, participants who viewed the influenza vaccine as safe and effective were more likely to be vaccinated against COVID-19.

### Hypothetical vaccine scenarios

The hypothetical scenarios reveal a strong willingness to accept vaccines for high-mortality diseases, with 86% of participants choosing to vaccinate for a “rare but deadly illness with 50% mortality.” This high acceptance reflects an understanding of the risk-benefit ratio in severe scenarios, with the perceived risk of a new illness being weighed against the potential risks of vaccination. While most participants selected to obtain both hypothetical vaccines, consistent with their COVID-19 and influenza vaccination choices, there was a slight shift when the rhetoric around risk changed. This willingness decreased to 73% for a “more common, less deadly illness with 1% mortality” highlighting the challenge of promoting vaccines for diseases perceived as less threatening. These findings suggest that communicating the severity and risks associated with COVID-19 and other diseases could enhance vaccine acceptance.

### Sources of vaccine information

Healthcare providers emerged as the most trusted source of vaccine information, with 90% of participants considering them trustworthy. This emphasizes the importance of involving healthcare professionals in vaccination campaigns and ensuring they are equipped with accurate and up-to-date information. Government sources and family and friends were moderately trusted, while social media was the least trusted source, indicating a potential area for improvement in public health messaging and combating misinformation.

## Limitations

Limitations of enrolling equal numbers of patients from each clinic include language barriers, variations in clinic volume, and patients utilizing multiple free clinics for care. Patient recruitment was additionally limited by restricted hours of clinic operation, limited enumerators, and survey administration being secondary to patient care.

Patient survey fatigue and varied interpretation in the setting of interpreters may affect response accuracy. Additionally, patients who rely on clinic staff for medical care may have hesitated to express their opinions, possibly introducing reporting bias. Limited coverage for languages beyond English, Spanish, Cantonese, and Vietnamese may have impacted translation and enumeration. Furthermore, using multiple enumerators may have introduced variance in survey delivery.

The results were analyzed and presented in the format of descriptive analysis due to the sample size. We analyzed all patients across clinics together as the patients were all un- or underinsured. Since most participants were amenable to vaccination, our sample size for the unvaccinated cohorts presented in smaller numbers, possibly making the results less generalizable.

## Conclusion

Free clinics have a mission to serve a specific demographic. Therefore, they recruit volunteers who are passionate about working with and intentionally building trust and respect within the communities that they serve through actions and outreach. Community-based, patient-centered care such as this fosters positive patient-provider relationships and promotes culturally responsive healthcare. Given the widespread vaccine hesitancy seen during the height of the COVID-19 pandemic, this study aimed to investigate perceptions of vaccines held by patients within free clinics that serve diverse communities. Notably, we found that the predominantly low-income population served by free clinics in the Greater Sacramento area had higher vaccination rates than the surrounding Sacramento County, reflecting a high level of trust placed in healthcare providers for vaccine information. These findings underscore clinics’ responsibility to centralize vaccine information and enhance accessibility. Continued vaccination efforts, monitoring patient responses and needs, and drawing insights from successful outreach models are imperative. The findings from this study contribute valuable insights to understanding diverse patients’ vaccine perceptions and can guide vaccination efforts at free/low-cost clinics serving under-resourced communities nationwide.
